# New solar energy-storage resource of plasmon-activated water solution with higher chemical potential

**DOI:** 10.1038/s41598-020-77815-3

**Published:** 2020-11-30

**Authors:** Chih-Ping Yang, Shih-Hao Yu, Fu-Der Mai, Tai-Chih Kuo, Yu-Chuan Liu

**Affiliations:** 1 Department of Biochemistry and Molecular Cell Biology, School of Medicine, College of Medicine, Taipei Medical University, No. 250, Wuxing St., Taipei, 11031 Taiwan; 2 Cell Physiology and Molecular Image Research Center, Wan Fang Hospital, Taipei Medical University, Taipei, Taiwan

**Keywords:** Energy science and technology, Nanoscience and technology

## Abstract

Nowadays, solar energy is the most environmentally friendly energy source to drive many chemical reactions and physical processes. However, the corresponding fabrication procedures for obtaining excellent energy-storage devices are relatively complicated and expensive. In this work, we report an innovative strategy on plasmon-activated water (PAW) serving as energy-storage medium from solar energy. The lifetime of the created energetic PAW solution from hot electron transfer (HET) on Au nanoparticles (AuNPs) illuminated with sunshine can last for 2 days, making the energy-storage system is practically available. Encouragingly, the energy-conversion efficiency from the solar energy in the PAW solution is ca. 6.7%. Compared to conventional deionized (DI) water solution, the prepared metastable PAW solution exhibited distinctly higher chemical potential at room temperature. It demonstrates abilities in faster evaporation and enhancing chemical reactions, including hydrogen evolution reaction (HER) and oxygen evolution reaction (OER). Our proposed strategy on the simple and cheap energy-storage system based on prepared PAW utilizing solar energy is the first time shown in the literature.

## Introduction

Liquid water is the most abundant liquid on the earth and is recognized as the most environmentally friendly solvent in chemical reactions. Water’s unique chemical and physical properties come from its flexible dynamic network of hydrogen bonds (HBs), in which HBs are broken and formed at equilibrium on a picosecond time scale^1,2^. Because investigating water’s local structure is still challenging^3,4^, well-known water properties are mainly based on free bulk water in spite of the original tetrahedral HB network being destroyed when water is confined in a nanosized environment or localized at interfaces^5,6^. These heterogeneous interactions result in corresponding changes in water’s HB-dependent properties^7,8^. In fact, water deviated from the tetrahedral symmetry structure of bulk water would create disordered defect structure. As a result, it can reduce the size of water clusters. Compared to bulk bound water clusters, disordered water clusters with weak HBs have more free water molecules which can interact with other species to enhance the activity. As reported by Velasco-Velez et al.^9^, analysis of an ab initio molecular dynamic (AIMD) simulation revealed that ~ 50% of interfacial water molecules lie flat on a gold (Au) surface with broken HBs, and this population of broken HB molecules is substantially higher than the 22% found in bulk water.

On the other hand, due to increasing shortages of available energy in the world, the harvesting of solar energy has drawn enthusiastic interest due to it being a more environmentally friendly energy conversion process. For practical use, solar energy can be converted to heat, electricity, and energy-rich chemicals (solar fuel). Conversion to electricity and fuel is the most interesting and valuable because these forms of energy are more convenient to use^10–13^. Correspondingly, energy-storage materials with enhanced energy-storage densities comprising highly stable devices are rapidly being developed. However, fabrication procedures for obtaining excellent energy-storage devices are relatively complicated. As shown in the literature, stable polymer phase-change materials embedded with Fe_3_O_4_-functionalized graphene nano-sheets were prepared serving as sunlight- and magnetic-driven energy conversion and storage nano-composites^14^. Also, to enable the synergistic coupling of electrochemical storage and light harvesting in a single electrode bias-free and solar-driven pseudocapacitors utilizing nanorod arrays of ZnO@NiO were proposed^15^.

Because Au nanoparticles (NPs) possess well-defined localized surface plasmon resonance (LSPR) bands in the ultraviolet and near-infrared regions, they are commonly utilized in fields of surface-enhanced Raman scattering (SERS)^16^, photothermal ablation of tumors^17^, and photochemical catalytic reactions^18^. Jia et al.^19^ reported the preparation of Au/CeO_2_ microsphere photocatalysts through an aerosol spray and a study of their photocatalytic activities toward the aerobic oxidation of 1-phenylethanol under visible light based on hot electron transfer (HET). Meanwhile, Yoo et al.^20^ showed that electromagnetized AuNPs in the presence of specific electromagnetic field (EMF) conditions facilitate the efficient direct lineage reprogramming to induce dopamine neurons in vitro and in vivo. However, these chemical and biochemical reactions of AuNP-based efficient energy transfer occur transiently. The unutilized energy at the moment from excited or electromagnetized AuNPs cannot be preserved, meaning that the produced energies are wasted. Water’s property and activity is critically dependent on the strength of hydrogen bonds (HBs) between water molecules. Innovative plasmon-activated water (PAW) was created utilizing HET on AuNPs with resonant illumination, as reported previously^21^. The created PAW with reduced HBs owns many distinct properties compared to bulk water. For examples, a smaller specific heat and a longer spin–lattice relaxation time. Recently, efficient and inexpensive catalysts are most developed for enhancing the efficiency on oxygen and hydrogen production. However, it is promising of an alternative approach to effective clean energy-relative reactions that employ PAW solutions, which are created from natural solar energy, with water molecules themselves possessing reduced HBs. The lifetime of HET is on the order of picoseconds, making the corresponding utilization limited and inconvenient. In this work, we suppose that the relatively large energetic barrier of HBs of bulk water could be overcome by utilizing solar-illuminated AuNPs to facilitate the dissociation of H_2_O. Also, we demonstrate that the PAW solution with a higher chemical potential preserved from solar energy can serve as a new energy-storage resource to enhance chemical reactions and physical processes.

## Results and discussion

### Distinct properties of prepared PAW

**Figure 1:**
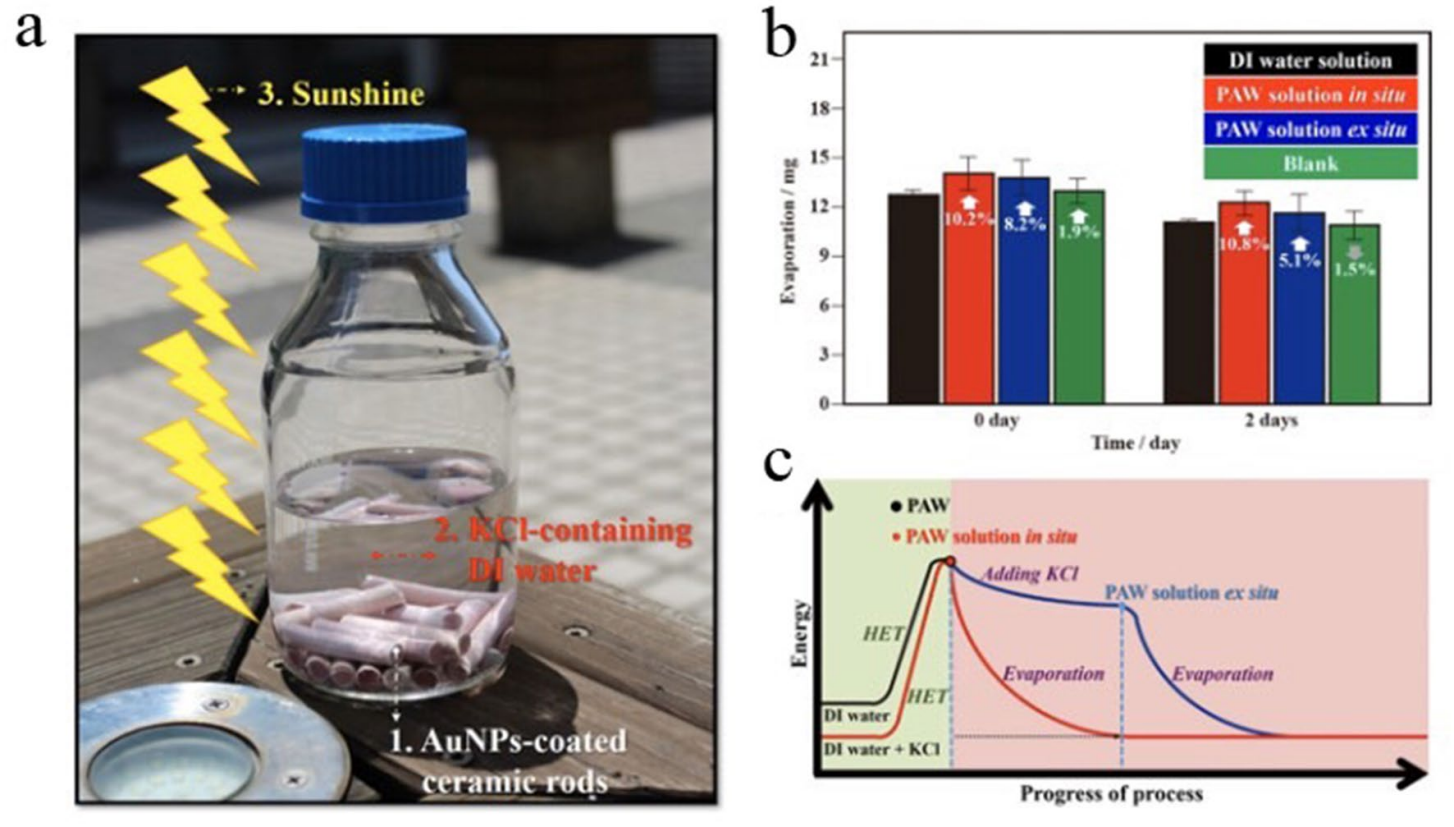
Reaction glass cell for creating plasmon-activated water (PAW) solutions under solar irradiation and corresponding evaporation rates of PAW-based solutions (0.1 M KCl) after exposure to sunlight for 3 h. Deionized (DI) water-based solutions (0.1 M KCl) are demonstrated for reference. (**a**) Gold nanoparticle (AuNP)-coated ceramic rods in glass sample vials with 0.1 M KCl-containing DI water solutions under solar irradiation: 1, AuNP-coated ceramic rods; 2, 0.1 M KCl-containing DI water; 3, sunshine. (**b**) Evaporation rates in 30 min of the as-prepared and aged (2 days) PAW-based, DI water-based, and blank experiment-based solutions (0.1 M KCl); the blank solution was obtained by using experimental conditions similar to those for preparing the PAW solution in situ but using blank ceramic rods with no AuNP coating. (**c**) Schematic descriptions of the energy-progress of the process curve of PAW solutions in energy transfer for the dissolution and evaporation processes.

From the viewpoint of thermodynamics, liquid water should be an excellent energy sink in utilizing solar energy, because its temperature is slowly raised when it is illuminated by sunshine and correspondingly being slowly decreased as it is cooled. However, most of the energy converted from solar energy is heat, and the dynamic equilibrium of HBs is on the level of picoseconds in water, making liquid water unavailable as an energy-storage resource when it is easily cooled to room temperature. On the other hand, HET can promote many chemical reactions, including the dissociation of hydrogen^22^ and activation of oxygen^23^. Interestingly, the dynamic processes of continuous and spontaneous forming, breaking, and rearranging of HBs occur at the picosecond scale, which is accordant with the lifetime of hot electrons^24^. Therefore, hot electrons may seize the moment to rapidly occupy breaking HBs and prevent them from reforming. This potential inspired us to utilize HET at excited AuNPs to create a metastable energetic PAW solution with intrinsically reduced HBs, which can serve as a new alternative energy-storage resource with a higher chemical potential from solar energy. Our previous study^21^ reported that PAW can be created utilizing HET on AuNPs with resonant illumination of green LEDs. In this work, natural sunshine with full wavelengths, including plasmon resonance wavelength for the same AuNPs, is utilized to produce the similar HET on AuNPs and the subsequent PAW. Figure 1a shows the reaction glass cells for creating the PAW solutions (0.1 M KCl) exposed to sunlight. The light red color of the AuNP-coated ceramic rods (originally white color) was observed. This color is the characteristic one of AuNPs with photocatalytic activity, which is similar with our previous report (AuNP-coated ceramic particles), showing the creation of PAW^21^. As shown in the literature^25,26^, cations and anions impact the electronic structure of liquid water, resulting in distortions of the geometrical arrangements of water molecules. Therefore, two kinds of PAW solutions were prepared to examine the influence of electrolytes on HBs of the produced PAW solutions under solar irradiation in the presence of AuNPs. The first was a PAW solution in situ, in which KCl electrolytes were added to DI water before solar irradiation. The second was a PAW solution ex situ, in which KCl electrolytes were added to DI water after solar irradiation. Water’s HBs are responsible for its fundamental abilities. Thus, this effect on the corresponding evaporation rate was first examined in ambient laboratory air. The left part of Fig. 1b demonstrates evaporation rates of different as-prepared aqueous solutions containing 0.1 M KCl. Based on the colligative properties of ionic solutions, the vapor pressure of ionic solutions should be lower than that of a pure solvent. Thus, all experiments were performed on a platform of an orbital shaker for 30 min to accelerate the evaporation process. The measured evaporated masses in 30 min were 12.73 ± 0.25 (12.5, 13.0 and 12.7), 14.03 ± 1.01 (15.2, 13.5 and 13.4), and 13.77 ± 1.27 (15.2, 11.4 and 14.7) mg based on three replicated experiments (the following average data were also obtained using three replicated experimental results) for the DI water solution, PAW solutions in situ and ex situ, respectively. Magnitudes of the evaporation rates of the PAW solutions in situ and ex situ were respectively higher by ca. 10.2% and 8.2%, compared to that of the DI water solution. The unique property of the high boiling point of water is ascribed to its strong HBs; thus, these increased evaporation rates observed in PAW solution systems with weak HBs are interesting. Moreover, the measured evaporation mass in 30 min was 12.97 ± 0.76 (13.5, 13.3 and 12.1) mg for the blank solution, which was obtained using similar experimental conditions (in the absence of AuNPs) as used to prepare the PAW solution in situ. This magnitude of the evaporation rate was slightly higher, by ca. 1.9%, compared to that of the DI water solution, indicating that HET for weakening the HBs of water only occurred in the presence of AuNPs under solar irradiation. Moreover, for sunshine-irradiated experiments performed in open bottles, magnitudes of the evaporation rates of the PAW solutions in situ were higher by ca. 17.5%, 15.3% and 9.8% in the first, second and third hours, respectively, compared to those of the blank solutions, as shown in Fig. S1. These results confirm that the phenomena are photochemical processes. As shown in the literature, the potential of water is proportional to the natural logarithm of its vapor pressure according to the following equation^27^:

1 $$ {\upmu } = {\upmu }_{{\text{o}}} + {\text{ RT ln}}\left( {{\text{f}}/{\text{f}}_{{\text{o}}} } \right) $$

where μ and μ_0_ (J mol^−1^) are defined as the potentials (i.e., thermodynamic activities or energies) at different vapor pressures of f and f_0_, respectively; R is the gas constant (8.314 J mol^−1^ K^−1^); and T is the temperature in K. Compared to the DI water solution, the higher evaporation rate of the PAW solution means that the PAW solution possesses a higher vapor pressure than that of the DI water solution at room temperature. This suggests that the metastable PAW solution possesses higher activity similar to those of energy-rich chemicals with reduced HBs due to HET, which is in accordance with the increased activity observed for confined water^28^. The right part of Fig. 1b demonstrates corresponding evaporation rates of different aqueous solutions containing 0.1 M KCl for 2 days after their preparation. In aging, the sealed glass sample bottles were placed in a dark atmosphere and in ambient laboratory air. The measured evaporation masses at 30 min were 11.07 ± 0.12 (11.2, 11.0 and 11.0), 12.27 ± 0.74 (11.7, 12.0 and 13.1), 11.63 ± 1.72 (10.9, 10.4 and 13.6), and 10.90 ± 0.87 (11.3, 9.9 and 11.5) mg for the DI water solution, the in situ and ex situ PAW solutions, and the blank solution, respectively. The magnitudes of the evaporation rates of the energetic PAW solutions in situ and ex situ were still higher by ca. 10.8% and 5.1%, respectively, compared to that of the DI water solution after aging for 2 days. Similarly, the evaporation rate of the blank solution was close to that of the DI water solution. Conversion of the solar energy stored in the PAW solution means that is can be utilized for a couple of days after its creation; thus, it can possibly serve as a new energy-storage resource. Of course, the activity of the created PAW will decay with time.

The effects of adding electrolytes to the water on the corresponding properties of the prepared PAW solutions are another interesting finding. The increased evaporation rates compared to the DI water solution were more significant for the PAW solution in situ as observed in both fresh and aged samples. These phenomena were independent of the heat of the solution because using KCl with a positive heat of solution (17 kJ mol^−1^) and using LiCl with a negative heat of solution (− 38 kJ mol^−1^) both demonstrated consistent results of higher evaporation rates in PAW solutions compared to them in DI water solutions, as shown in our previous study^29^. For the PAW solution ex situ, the reduced evaporation rate, compared to the PAW solution in situ, can be ascribed to the decreased vapor pressure due to the well-known colligative properties of ionic solutions, when KCl was added to the PAW. Some of the conserved energy from solar irradiation is utilized as the heat of solution for the dissolution of KCl in PAW. This release of chemical energy was accompanied with the formation of HBs in water molecules. Therefore, a new metastable PAW solution ex situ was formed with lower energy and stronger HBs compared to the PAW solution in situ. For the PAW solution in situ, the energy lost in dissolving KCl in DI water can easily be supplemented from HET under solar irradiation for 3 h. Therefore, the PAW solution in situ is in a metastable state with a higher energy compared to the PAW solution ex situ. Pure PAW without electrolytes is in the same metastable state as the PAW solution ex situ under the same solar irradiation. The corresponding energy-progress process of the PAW solutions is illustrated in Fig. 1c. Throughout this work, unless stated otherwise, aqueous solutions were prepared with 0.1 M KCl.

Moreover, similar photochemical experiments, as the one shown in Fig. 1a, were performed to demonstrate the phenomenon of HET based on electrolyte-free water. The measured zeta potentials of PAW were − 29.6 ± 0.56 and − 26.9 ± 0.73 mV, respectively, 0 and 2 days after its creation; these values are close to electronically neutral DI water (− 1.63 ± 0.41 and − 1.58 ± 0.29 mV, respectively, 0 and 2 days after its preparation). Meanwhile, compared to DI water, the intensities of hydroxyl free radicals measured by electron spin resonance spectroscopy^21^ decreased by 45 ± 3.9% and 29 ± 3.3%, respectively, for as-prepared and 2-day-aged PAW.

**Figure 2:**
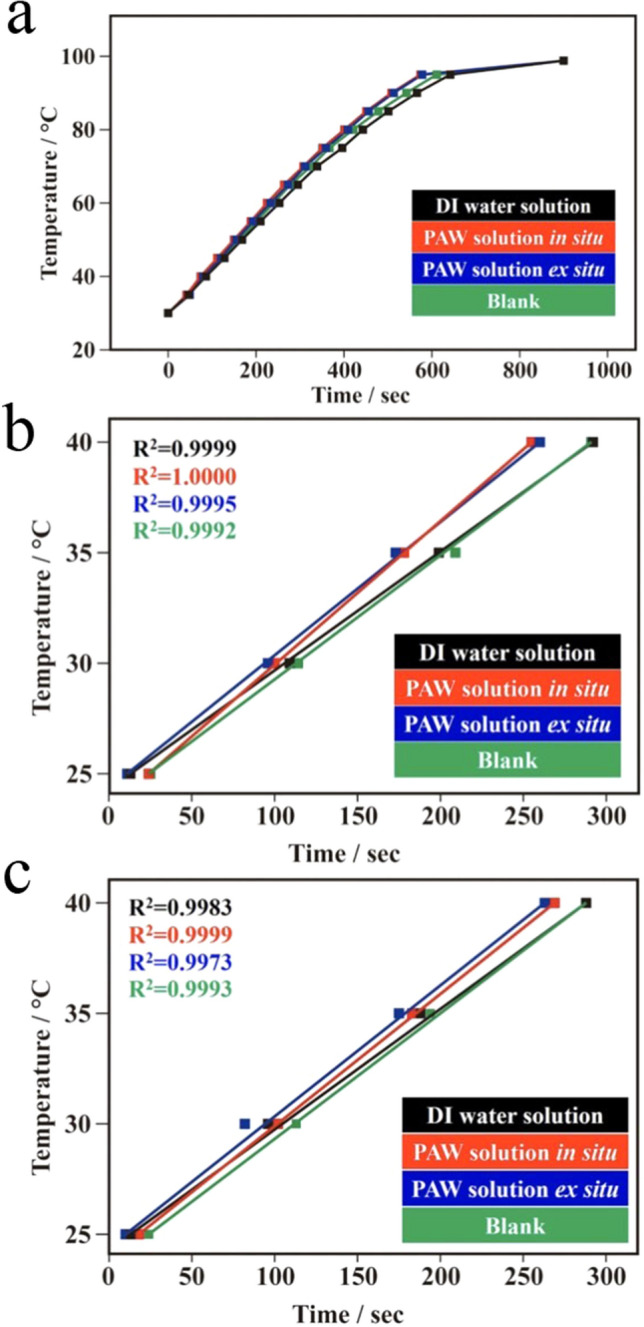
Specific heat (compared to the 0.1 M KCl deionized (DI) water solution) of plasmon-activated water (PAW)-based solutions (0.1 M KCl) after exposure to solar irradiation for 3 h. (**a**) Rates of rising temperatures measured in the as-prepared PAW-based and DI water-based solutions (0.1 M KCl) with the same masses under a constant applied power. (**b**) The temperature-heating time dependence between room temperature and 40 °C of the as-prepared PAW-based and DI water-based solutions. (**c**) The temperature-heating time dependence between room temperature and 40 °C of aged (for 2 days) PAW-based and DI water-based solutions. The blank solution was obtained using experimental conditions similar to those for preparing the PAW solution in situ but using blank ceramic rods without an AuNP coating.

Calculating evaporation rates also disclosed a difference in HBs of the water solutions, which indicated different heat capacities. As expected, temperatures of both 0.1 M KCl-containing solutions increased with the heating time (Fig. 2a). At temperatures of > 90 °C, the lines became flattened because the water was nearly boiling. The boiling point was 97.4 ± 0.2 °C for the DI water solution; while they were respectively reduced to 96.6 ± 0.1 and 96.4 ± 0.2 °C for the PAW solutions in situ and ex situ. Similarly, the boiling point of the blank solution (97.8 °C) was close to that of the DI water solution. It is recognized that HBs serve as a storehouse of energy. Instead of directly raising the temperature of the solution, part of heat is utilized for breaking HBs of water molecules. Due to fewer HBs remain at higher temperatures, thus, the heat capacity would decrease as the temperature increasing. In contrast, it would increase with rising temperatures. This phenomenon suggests that the added heat was utilized for breaking HBs of water molecules also for raising the temperature. Particularly, maintaining the degree of freedom between water molecules to prevent them from re-bonding. As a result that the PAW solutions can own intrinsically reduced HBs and could further prevent the re-bonding of HBs between water molecules. Therefore, it reduces the energy gap as the temperature rising. The specific heats of the PAW solutions in situ and ex situ were between 25 and 40 °C, which respectively demonstrate reduced values of 0.832 and 0.889 compared to the heat capacity of the DI water solution which was set to 1 (calculated from Fig. 2b). Because all of the experiments, using the same mass of water, were performed on the same heater with a constant heating rate the specific heats were inversely proportional to the slopes of lines, as shown in Fig. 2b. Compared to a general difference in specific heats of < 1% for DI water at low and high temperatures^30^, the 17% difference was indeed significant. This magnitude of the specific heat was slightly reduced by ca. 3.7% for the blank solution, compared to that of the DI water solution. These results are in agreement with the correlation of the water cluster size and heat capacity, in which the heat capacity of (H_2_O)_21_ is smaller than that of (H_2_O)_50_ at 27 °C^31^. After aging for 2 days, the specific heats of the PAW solutions in situ and ex situ were between 25 and 40 °C, which respectively demonstrated reduced values of 0.909 and 0.929 compared to the heat capacity of the DI water solution which was set to 1 (calculated from Fig. 2c). The magnitudes of the specific heats of the energetic PAW solutions in situ and ex situ were still reduced by ca. 9.1% and 7.1%, respectively, compared to that in the DI water solution after aging for 2 days.

**Figure 3:**
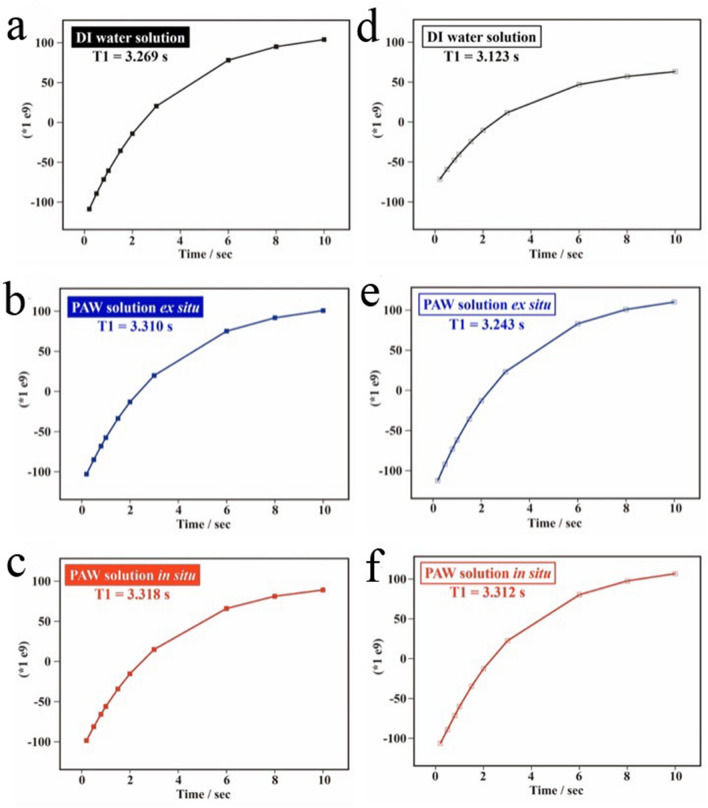
NMR-*T*_1_ represents the time required for the longitudinal component of magnetization to recover to its equilibrium value after applying a perturbing pulse sequence. Spectra represent spectral signals as a function of the repetition time for (**a**) the deionized (DI) water solution (0.1 M KCl, as-prepared), (**b**) the plasmon-activated water (PAW) solution ex situ (0.1 M KCl, as-prepared), (**c**) the PAW solution in situ (0.1 M KCl, as-prepared), (**d**) the DI water solution (0.1 M KCl, aged for 2 days), (**e**) the PAW solution ex situ (0.1 M KCl, aged for 2 days), and (**f**) the PAW solution in situ (0.1 M KCl, aged for 2 days).

The evidence for weak HBs within pure PAW was discussed with deconvoluted Raman spectra in O–H stretching vibrations in our previous report^23^. These O–H stretching vibrations in Raman spectra are also sensitive to solutes in the water. Thus, the evidence for reduced HBs in PAW solutions was examined by diffusion averages of the nuclear magnetic resonance (NMR) relaxation time (*T*_1_) in this work (Fig. 3). The strength of HB interactions among water molecules affected the spin–lattice relaxation time, *T*_1_, which represents the time required for the longitudinal component of magnetization to recover its equilibrium value after application of a perturbing pulse sequence. Under magnetic field fluctuations for as-prepared solutions, *T*_1_ values of the PAW solutions in situ and ex situ were 3.315 ± 0.004 and 3.281 ± 0.041 s, respectively, which were significantly longer than 3.230 ± 0.055 s of the DI water solution. Similarly, with solutions aged for 2 days, *T*_1_ values of the PAW solutions in situ and ex situ were 3.290 ± 0.032 and 3.240 ± 0.004 s, respectively, which were still longer than 3.139 ± 0.022 s of the DI water solution. These analyses of NMR relaxation times suggested an intrinsic reduction of HB structures in the PAW solution. These energy-rich chemicals of reduced HBs show promise as an energy-storage resource for PAW solutions.

### Distinctly electrochemical performance of prepared PAW

**Figure 4:**
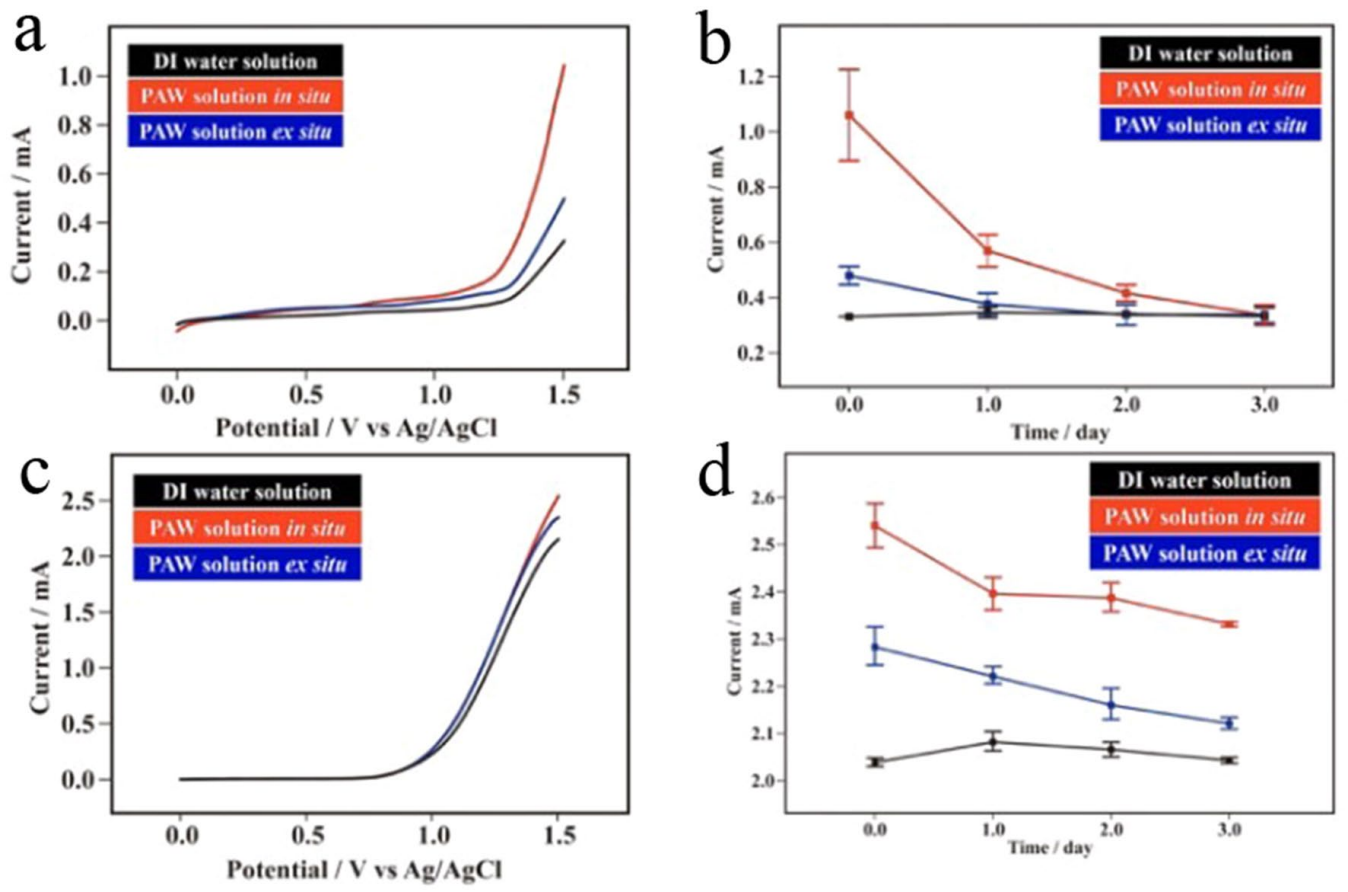
Linear sweep voltammetry (LSV) recorded on a planar Pt electrode for the oxygen evolution reaction (OER) in plasmon-activated water (PAW)-based and deionized (DI) water-based solutions. (**a**) LSV at scan rates of 0.05 V s^–1^ in the PAW solution in situ, the PAW solution ex situ, and the DI water solution (all containing 0.1 M KCl). (**b**) OER currents at 1.5 V vs. Ag/AgCl in the PAW solution in situ, the PAW solution ex situ, and the DI water solution (all containing 0.1 M KCl) for 0, 1, 2, and 3 days after their preparation. (**c**) LSV at scan rates of 0.05 V s^–1^ of the PAW solution in situ, the PAW solution ex situ, and the DI water solution (all containing 0.1 M NaOH). (**d**) OER currents at 1.5 V vs. Ag/AgCl in the PAW solution in situ, the PAW solution ex situ, and the DI water solution (all containing 0.1 M NaOH) for 0, 1, 2, and 3 days after their preparation.

Water molecules exist in the form of water clusters in bulk water due to strong HB interactions. It was reported that the interaction energy of H_3_O^+^–OH^−^ is 46.9 kJ mol^−1^, and it increases approximately 2.5-times when H_3_O^+^ associates with an additional four water molecules^32^. Based on these facts, creating PAW solutions with markedly weaker HB interactions could be advantageously used for efficient water splitting, compared to conventional DI water solutions. Potential applications of PAW solutions in OERs and HERs were evaluated by electrochemical linear sweep voltammetry (LSV). Figure 4a demonstrates the corresponding results of OERs in PAW-based and DI water-based solutions (0.1 M KCl). The onset potentials for the PAW solutions were markedly smaller (cathodic shifts) than that for the DI water solution, especially for the PAW solution in situ. This indicates that the required electrolytic energy for the OER was indeed reduced via weakening water’s HBs. As the applied potential exceeded the onset potential, the current distinctly increased. At a vertex of 1.5 V, the respective recorded currents were 1.060 ± 0.165 and 0.479 ± 0.033 mA for the PAW solutions in situ and ex situ, which were ca. 220% and 40% higher than the 0.331 ± 0.005 mA for the DI water solution. As shown in the literature, to increase the efficiency of OERs, the most common approaches have focused on new and cheap catalysts with different chemical compositions and structures^33,34^. Investigations of the effect of the structure of reactant water itself on the corresponding efficiency of OERs have been less reported in the literature. Moreover, the increased efficiencies (depending on the recorded currents) of OERs performed in PAW solutions decreased with storage time (Fig. 4b). Similar currents for as-prepared DI water solutions and those aged for 3 days mean that the HB structure in DI the water solution was stable during storage. However, the phenomenon of decreasing efficiencies for PAW solutions suggests that the broken HBs within water molecules recombine over time, resulting in a reduction in the efficiency of electrolytic water splitting. For the PAW solution ex situ, this increased efficiency in the OER was slight after aging for 1 day. Interestingly, for the PAW solutions in situ, these increased efficiencies in OERs of ca. 64% and 22% in magnitude were still significant after aging for 1 and 2 days, respectively. Again, as shown in Fig. S2, this increased efficiency of OERs for the as-prepared blank solution was slightly higher by ca. 9.2%, compared to that for the DI water solution, indicating that HET for weakening HBs of water only occurred in the presence of AuNPs under solar irradiance. Figure 4c,d demonstrate the corresponding OERs performed in alkaline solutions containing 0.1 M NaOH. Comparing Fig. 4c showing experiments performed in alkaline solutions with Fig. 4a showing experiments performed in neutral solutions, it was found that the recorded currents in alkaline solutions were significantly larger than those in neutral solutions. This is reasonable because an alkaline solution is favorable for OERs. At a vertex of 1.5 V, the recorded currents were 2.5400 ± 0.1949 and 2.2830 ± 0.2621 mA for the PAW solutions in situ and ex situ, respectively, which were ca. 25% and 12% higher than the 2.0386 ± 0.1708 mA of the DI water solution. Interestingly, for the PAW solutions in situ and ex situ, these respective increased efficiencies in OERs were of ca. 14% and 3.8% after aging for 3 days, as shown in Fig. 4d. This increase was still significant for the PAW solution in situ.

**Figure 5:**
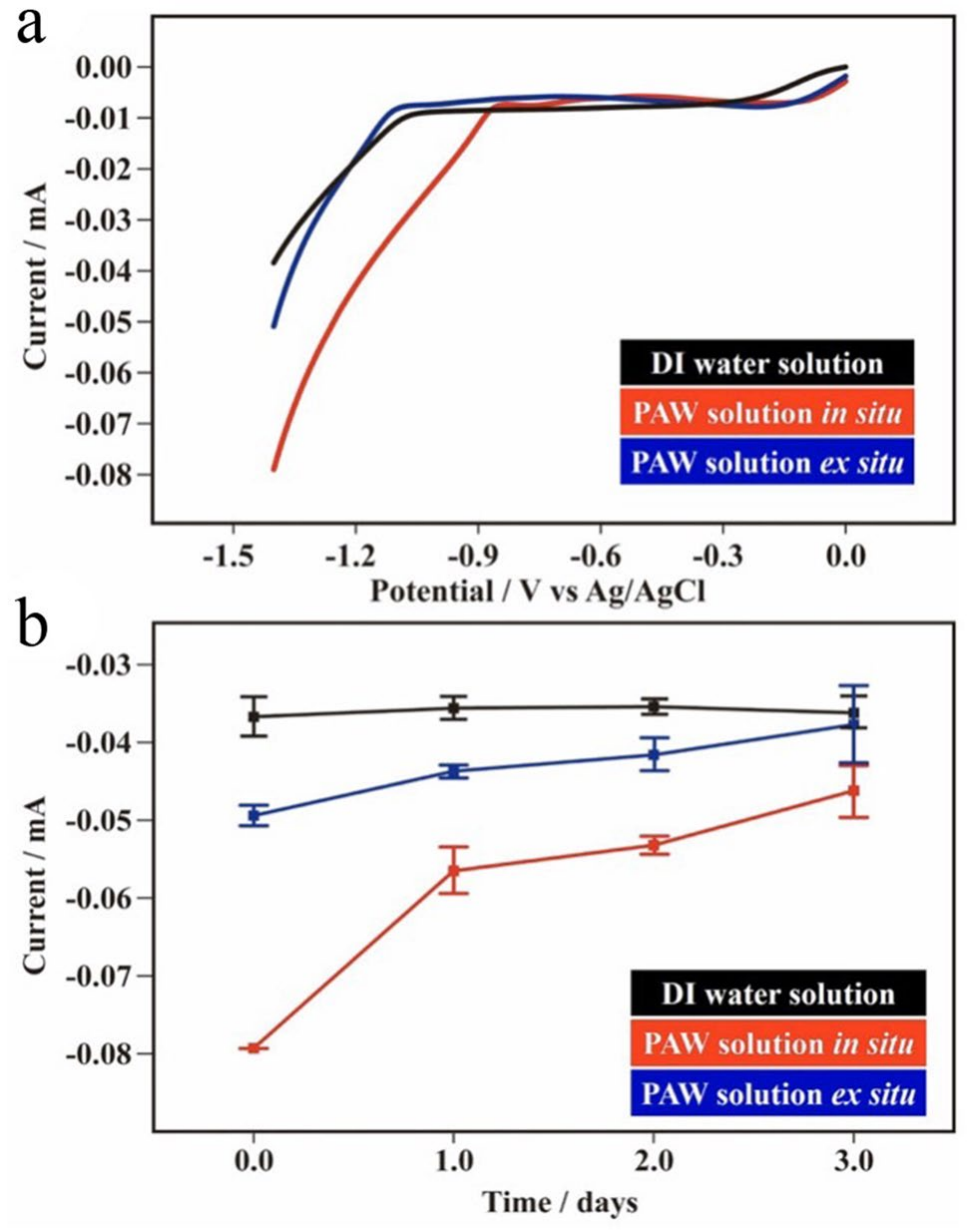
Linear sweep voltammetry (LSV) recorded on a planar Pt electrode for the hydrogen evolution reaction (HER) in plasmon-activated water (PAW)-based and deionized (DI) water-based solutions (0.1 M KCl). (**a**) LSV at scan rates of 0.05 V s^–1^ in the PAW solution in situ, the PAW solution ex situ, and the DI water solution. (**b**) HER currents of at − 1.4 V vs. Ag/AgCl in the PAW solution in situ, the PAW solution ex situ, and the DI water solution for 0, 1, 2, and 3 days after their preparation.

Similarly, Fig. 5a shows the corresponding results of HERs in PAW-based and DI water-based solutions (0.1 M KCl). Onset potentials of the PAW solutions exhibited significant anodic shifts compared to the DI water solution, especially for the PAW solution in situ. At a vertex of − 1.4 V, the recorded currents were − 0.0793 ± 0.0006 and − 0.0494 ± 0.0013 mA for the PAW solutions in situ and ex situ, respectively, which were ca. 120% and 35% higher than the − 0.0367 ± 0.0015 mA of the DI water solution. As expected, the increased efficiencies (depending on the recorded currents) of HERs performed in the PAW solutions decreased with the storage time (Fig. 5b). Similar currents for the as-prepared DI water solution and that aged for 3 days mean that the HB structure in DI water solutions was stable during storage. For the PAW solution ex situ, this increased HER efficiency was slight after aging for 2 days. Interestingly, for the PAW solutions in situ, these increased efficiencies in HERs of ca. 60%, 50% and 28% in magnitude were still significant after aging for 1, 2, and 3 days, respectively. Correspondingly, experimental results of evaporation rates, specific heats, OERs, and HERs suggest that the created metastable PAW solution in situ can serve as a new energy-storage material with energy-rich chemicals of reduced HBs.

**Figure 6:**
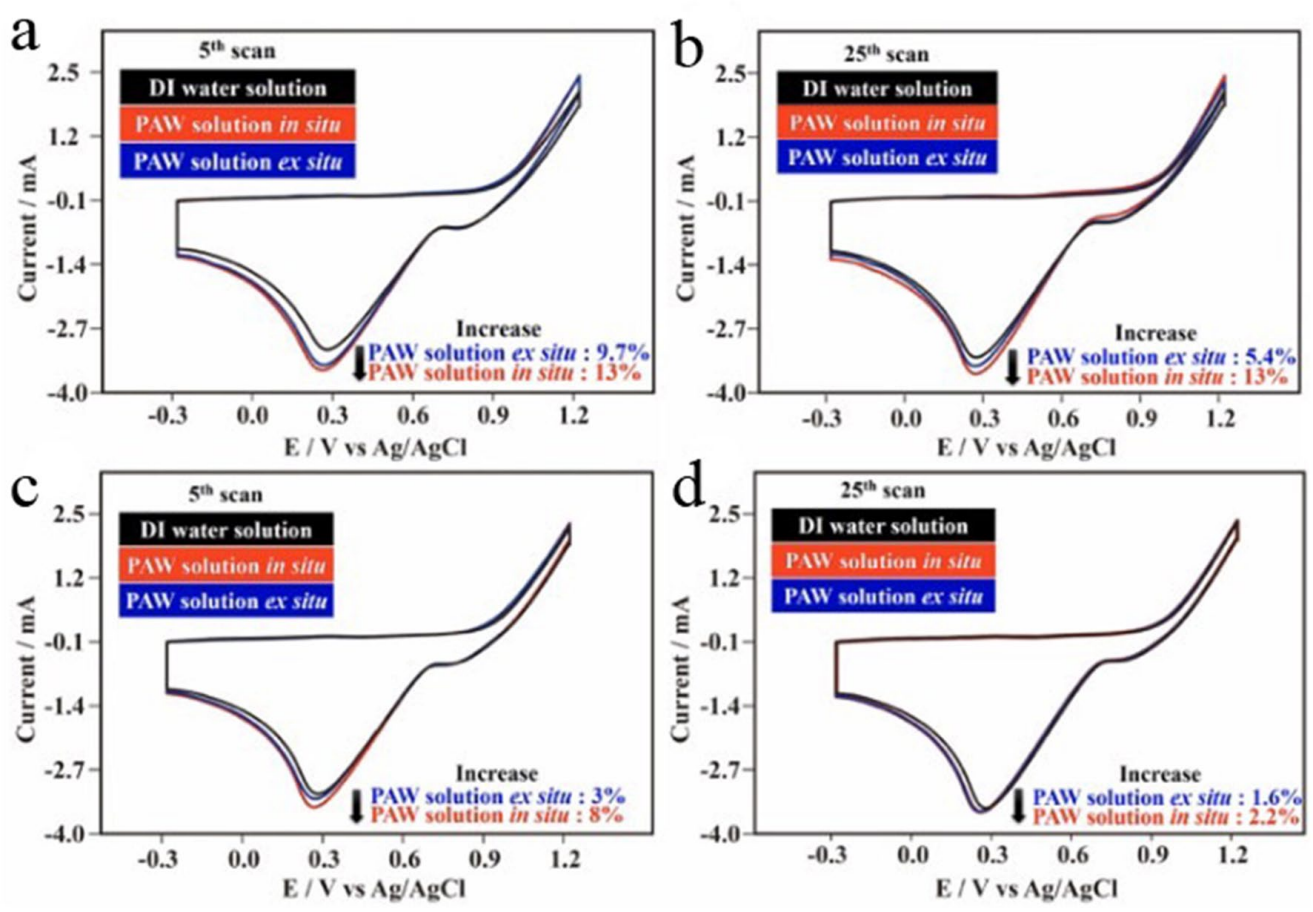
Cyclic voltammogram (CV) recordings on the same planar Au electrode showing the 5th (at the beginning) and the 25th (at the end) scans with oxidation–reduction cycle (ORC) treatments at 0.5 V s^–1^ to roughen the Au electrodes in plasmon-activated water (PAW)-based and deionized (DI) water-based solutions (0.1 M KCl). (**a**) The 5th scans in the as-prepared solutions; (**b**) the 25th scans in the as-prepared solutions; (**c**) the 5th scans in the solutions aged for 2 days; and (**d**) the 25th scans in solutions aged for 2 days.

**Figure 7:**
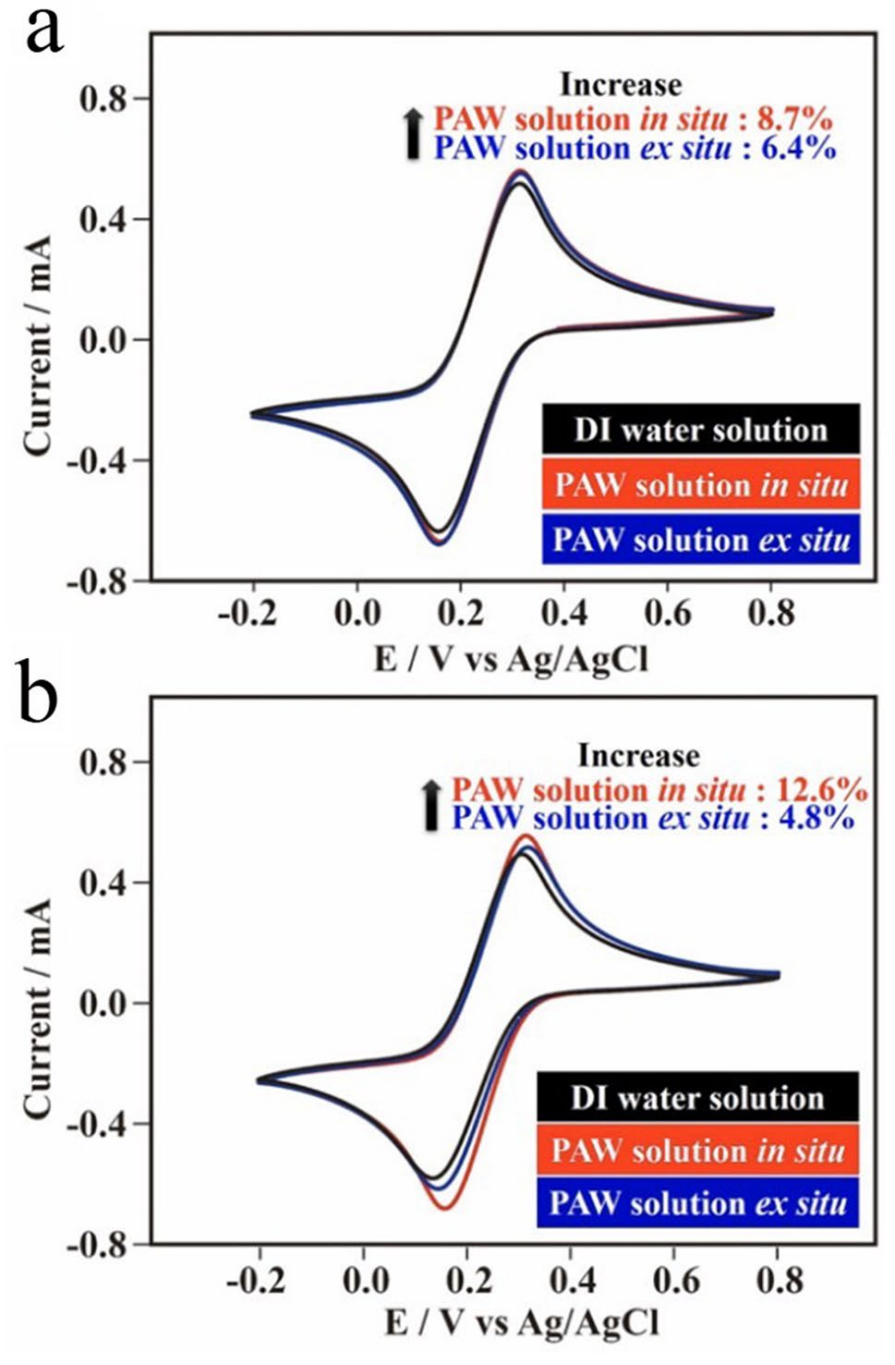
Cyclic voltammogram (CV) recordings on the same planar Au electrode showing the 3rd scans of the oxidation–reduction cycle (ORC) at 0.5 V s^–1^ in plasmon-activated water (PAW)-based and deionized (DI) water-based solutions (50 mM K_3_Fe(CN)_6_). (**a**) For the as-prepared solutions and (**b**) for solutions aged for 2 days.

**Figure 8:**
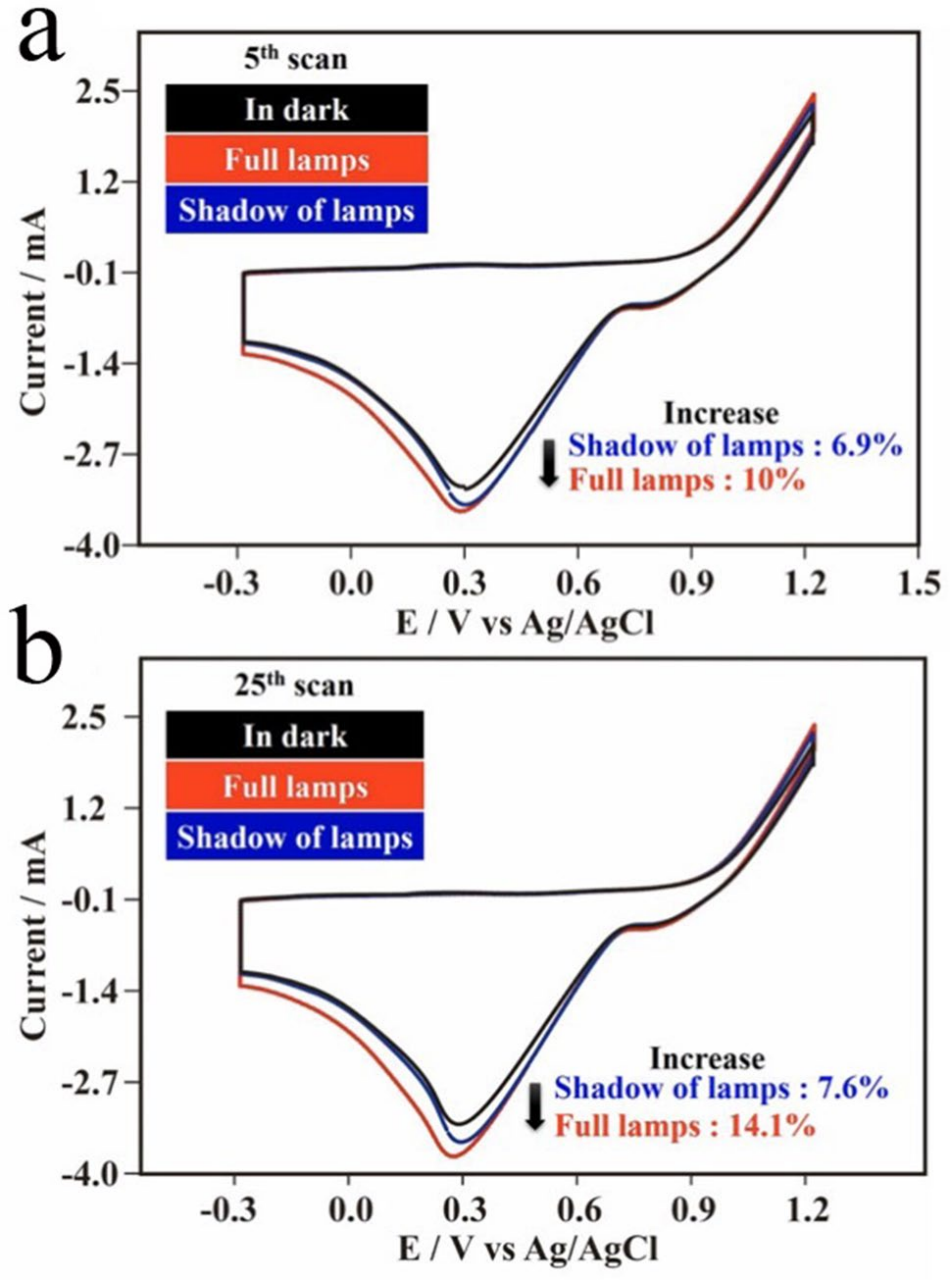
Cyclic voltammogram (CV) recordings of the same planar Au electrode showing the 5th (at the beginning) and the 25th (at the end) scans of the oxidation–reduction cycle (ORC) treatments at 0.5 V s^–1^ for roughening the Au electrode in deionized (DI) water-based solutions (0.1 M KCl) under different degrees of illumination from indoor fluorescent lamps. (**a**) The 5th scans in a completely dark condition, with full fluorescent lamps, and in the shadow of fluorescent lamps. (**b**) The 25th scans in a completely dark condition, with full fluorescent lamps, and in the shadow of fluorescent lamps.

Figure 6a demonstrates typical triangular voltammetric curves in the 5th scan for anodic dissolution and cathodic redeposition of Au respectively onto Au substrates in an as-prepared PAW solution in situ, an as-prepared PAW solution ex situ, and an as-prepared DI water solution for reference. Basically, the anodic dissolution and cathodic redeposition of AuNPs on substrates were easier in PAW solutions (a reflection of the enhanced current), especially in the PAW solution in situ, than in the DI water solution. In the oxidation–reduction cycle (ORC) treatment for roughening the Au substrate, AuNPs were deposited on the Au substrate. Intrinsic activation energy is necessary for this electrochemical reaction. In our previous study^21^, it was proposed that the chemical potential of PAW is higher than that of DI water. Because the PAW solution is energetic, the actual required activation energy was correspondingly reduced, compared to the DI water solution. Therefore, enhanced currents were obtained in the PAW solutions. Compared to the DI water solution, the cathodic redeposition currents at ca. 0.28 V vs. Ag/AgCl respectively increased by 13% and 9.7% for the PAW solutions in situ and ex situ. With up to 25 scans, as shown in Fig. 6b, these increases in cathodic redeposition currents at ca. 0.28 V vs. Ag/AgCl were similar. Compared to the DI water solution, the cathodic redeposition currents respectively increased by 13% and 5.4% for the PAW solutions in situ and ex situ. Similarly, as shown in Fig. 6c (the 5th scan) and 6d (the 25th scan) for solutions aged for 2 days, smaller increased currents were observed in experiments performed in the PAW solutions, especially in the PAW solution in situ, compared to the DI water solution. Other experimental results (Figs. 1, 2, 3, 4, 5 discussed before, and Figs. 7, 8 discussed later) all indicate that the property differences between the PAW solution and the DI water solution after aging for 2 days are significant. Therefore, this small difference in increment drops to around 2.2%, as Fig. 6d, most likely can be ascribed to the solution difference. This also suggests that the PAW solution in situ is suitable for serving as an energy-storage resource. As described in the experimental section, the electrolytes of KCl were added in water before and after the creations of PAW for the PAW solution in situ and the PAW solution ex situ, respectively. Additional energy is necessary for the dissolution of KCl in water. Thus, the potential energy of the intrinsically energetic PAW is reduced for the preparation of the PAW solution ex situ. This results in the enhanced current being correspondingly reduced, as compared to the PAW solution in situ. The higher currents at cathodic peaks observed in PAW solutions may be also ascribed to the higher diffusion coefficient and the higher electron transfer rate constant for electrochemical experiments performed in the PAW solution systems, as discussed below.

Figure 7a shows the CVs of K_3_Fe(CN)_6_ in the as-prepared PAW solutions and the as-prepared DI water solution. It can be observed that both the anodic and cathodic peak currents based on the PAW solutions were the highest ones, especially for the PAW solution in situ. Similarly, the potential energy of the intrinsically energetic PAW would be reduced in the dissolution of electrolytes for the preparation of the PAW solution ex situ. Thus, this results in the enhanced currents being correspondingly decreased, as compared to the PAW solution in situ. In accordance with the Randles–Sevcik equation^35^, the peak current is proportional to the square root of the diffusion coefficient. The calculated diffusion coefficients of K_3_Fe(CN)_6_ in the PAW solution in situ (2.34 ± 0.01 × 10^−6^ cm^2^ s^−1^) and in the PAW solution ex situ (2.27 ± 0.01 × 10^−6^ cm^2^ s^−1^) were ca. 17% and 14%, respectively, higher than that in the DI water solution (2.00 ± 0.01 × 10^−6^ cm^2^ s^−1^). These results suggest that the PAW solution has a function of enhancing the diffusion ability of species in water. The reason might be attributed to the reduced size of hydrated Fe(CN)_6_^3−/4−^ in the water. Within the DI water solution, hydration is associated with large water clusters due to the strong HB network of water. Contrarily, breaking the HB structure can reduce the size of water clusters in the PAW solution, resulting in higher mobility of Fe(CN)_6_^3−/4−^–H_2_O. Figure 7b shows the corresponding CVs of K_3_Fe(CN)_6_ in aged solutions. Also, the diffusion coefficients of K_3_Fe(CN)_6_ in the in situ PAW solution (2.32 ± 0.06 × 10^−6^ cm^2^ s^−1^) and in the PAW solution ex situ (1.99 ± 0.04 × 10^−6^ cm^2^ s^−1^) were ca. 25% and 7%, respectively, still higher than that in the DI water solution (1.86 ± 0.03 × 10^−6^ cm^2^ s^−1^) after the samples had aged for 2 days. Moreover, as discussed in the supporting information (SI, Figs. S3–S5), electron transfer rate constants (*k*_*s*_) of K_3_Fe(CN)_6_ in the PAW solution in situ (0.233 ± 0.014 s^−1^) and in the PAW solution ex situ (0.226 ± 0.015 s^−1^) were ca. 6.9% and 3.7%, respectively, higher than that in the DI water solution (0.218 ± 0.014 s^−1^). Also, these constants for the PAW solution in situ (0.257 ± 0.008 s^−1^) and for the PAW solution ex situ (0.233 ± 0.005 s^−1^) were still ca. 18% and 7.4%, respectively, higher than that for the DI water solution (0.217 ± 0.005 s^−1^) after the samples had aged for 2 days. Moreover, as discussed in Fig. 7, including Figs. S3–S5, the higher diffusion coefficients and the higher electron transfer rate constants for experiments performed in PAW-based solutions contributed to the corresponding more-efficient OERs and HERs. In this work, the energy from the solar energy conversion is stored in the PAW solutions. This concept is quite different from the conventional situation. Two days later, the PAW solution can be retreated with the same process to again become an energetic PAW solution. That means that the PAW solution can be recycled.

The energy-conversion efficiency, η, in the preparation of a PAW solution under illumination of sunshine was estimated from the ratio of the energies required for breaking the HBs of bulk water and for raising the temperature of the PAW solution to that provided by solar energy, as defined below.

2 $$ {\upeta } = \, ({\text{E}}_{{{\text{HB}}}} {\text{M}}_{{{\text{water}}}} + {\text{ ms}}\Delta {\text{T}})/ \, ({\text{P}}_{{{\text{sunshine}}}} {\text{t}}) \times {1}00 \, \% $$

where the energy of hydrogen bonds, E_HB_, of 20 kJ mol^−1^ was used. To obtain 250 g (or 250 cm^3^, when using a density of 1 g cm^−3^) of a PAW solution the moles of bulk water, M_water_, in which hydrogen bonds were broken, were calculated from the moles (14 mol) multiplied by the difference in values of DNHBW (degree of non-hydrogen-bonded water) of DI water (21.29%) and PAW water (26.23%)^21^ under illumination by sunshine. The sunshine power (P_sunshine_) of 1 kW m^−2^ was used^36^ and the 3-h illumination time, t, is 10,800 s. The surface area of a 500 mL-glass bottle is ca. 0.035 m^2^. The mass of the PAW solution is 250 g. A specific heat, s, of 0.0042 kJ g^−1^ °C^−1^ is used for the PAW solution. The measured temperature difference, ΔT, is 11 °C. Therefore, the energy-conversion efficiency of the preparation of the PAW solution under the illumination of sunshine was approximately 6.7% ((13.8 + 11.6) kJ/378 kJ). Excluding the obtained sensible heat the energy-conversion efficiency from the solar energy conversion in the PAW solution is ca. 3.7%. Moreover, in Eq. (2), E_HB_M_water_ and msΔT are equivalent to the required energies on the latent and sensible heats, respectively. The required latent heat is roughly proportional to the increased evaporation rate of PAW compared to DI water. As discussed before in Fig. S1, for sunshine-irradiated experiments performed in open bottles, magnitudes of the evaporation rates of the PAW solutions in situ were higher by ca. 17.5%, 15.3% and 9.8% in the first, second and third hours, respectively, compared to those of the blank solutions. Based on the calculated E_HB_M_water_ of 13.8 kJ for 3-h sunshine-irradiated experiment, the estimated E_HB_M_water_ are ca. 5.67 kJ (13.8 × (17.5%/(17.5 + 15.3 + 9.8)%)) and 10.6 kJ (13.8 × ((17.5 + 15.3)%/(17.5 + 15.3 + 9.8)%)) for 1-h and 2-h sunshine-irradiated experiments, respectively. Also, the measured temperature differences, ΔT, are 4.3 and 8.4 °C for 1-h and 2-h sunshine-irradiated experiments, respectively. Thus, the energy efficiencies for the preparations of the PAW solutions under the illumination of sunshine are ca. 8.1% ((5.67 + 4.52) kJ/126 kJ) and 7.7% ((10.6 + 8.82) kJ/252 kJ) for the 1-h and 2-h sunshine-irradiated experiments, respectively. Excluding the obtained sensible heats the energy-conversion efficiencies from the solar energy conversions in the PAW solutions are ca. 4.5% and 4.2% for 1-h and 2-h sunshine-irradiated experiments, respectively. The energy-conversion efficiency uniquely developed in this work based on a water solution is comparable to other complicated systems, like the bifunctional NiFeSP/NF electrocatalyst implements unassisted solar-driven water splitting with a solar-to-hydrogen conversion efficiency of ∼ 9.2%^37^. It is also comparable to that of the TiO_2_/dots/hibiscus/CdS photoanode with the poly(3,4-ethylenedioxypyrrole) @MnO_2_ counter electrode in an aqueous polysulfide–silica gel electrolyte delivers a power conversion efficiency of 6.11%^38^.

PAW can be created for water on AuNPs under resonant illumination^21^. In this work, an energetic PAW solution was obtained from HET of AuNPs excited by solar irradiation. Actually, this effect could also be observed on the rough surface of the Au substrate with AuNPs obtained from the ORC procedure under illumination from indoor fluorescent lamps. Figure 8a demonstrates the corresponding CV curves in the 5th scans for anodic dissolution and cathodic redeposition of Au, respectively, onto Au substrates in DI water with 0.1 M KCl under different degrees of illumination. Basically, the anodic dissolution and cathodic redeposition of AuNPs on substrates were easier in DI water solutions (a reflection of enhanced currents) with illumination, especially for more-powerful illumination with fluorescent lamps with no shadows on the substrate, than in a fully dark condition. Compared to the fully dark condition, cathodic redeposition currents at ca. 0.30 V vs. Ag/AgCl respectively increased by 10% and 6.9% for the environments with full illumination and with shadows on the substrate. With up to 25 scans, as shown in Fig. 8b, these increases in cathodic redeposition currents at ca. 0.30 V vs. Ag/AgCl were similar but more significant because more AuNPs were deposited on the substrate with the increase in scanning. Compared to the fully dark condition, the cathodic redeposition currents respectively further increased by 14.1% and 7.6% for the environments with full illumination and with shadows on the substrate because more AuNPs were available for HET at higher scans.

The experimental results discussed above support that the relatively large energetic barrier of HBs of bulk water can be overcome by utilizing solar-illuminated AuNPs to facilitate the dissociation of H_2_O. Moreover, the created PAW solution with a higher chemical potential preserved from solar energy can serve as a new energy-storage resource to enhance water-related chemical reactions (like oxygen reduction reaction and production of H_2_O_2_ from water) and physical processes (like efficient water evaporation in desalination). This also resolves the concerning issue of the effective utilization of solar-driven HET with picosecond lifetimes.

In summary, we have successfully utilized the HET of solar energy-excited AuNPs to prepare PAW solutions with energy-rich chemicals as a new energy-storage resource for further chemical reaction. The PAW-based energy-storage system is simple and practical. This developed strategy of effectively utilizing transient HET makes relative applications more convenient. The energy storage efficiency of the solar energy conversion of the PAW solution is ca. 6.7%, which is comparable to other complicated systems shown in the literature. Compared to conventional DI water, the activity of the created energetic PAW solution can last for 2 days. The resulting metastable PAW solutions exhibited their ability to enhance HERs and OERs in fields of green energies. In particular, for the PAW solution in situ system, enhanced OER efficiencies of ca. 220%, 64%, and 22% were measured for the as-prepared solution, and solutions aged for 1 and 2 days, respectively. These findings of a metastable PAW solution in situ with distinct activity for chemical reactions at room temperature, like energy-rich chemicals, are first presented in the literature. Further innovative applications of PAW in water-related fields and development of strategies for maintaining distinct activities of PAW are worthy of studies in the future.

## Methods

### Preparation of AuNP-coated ceramic rods

Twenty rinsed ceramic rods (with a diameter of 0.8 cm and a length of 4 cm, also see Supporting Information (SI) for their components) were immersed in a sealed glass container with a 200-mL solution containing 50 ppm AuNPs (ca. 10 nm) in the dark for 1 day (see SI for the detailed preparation of AuNPs). After this process the AuNP concentration was reduced to ca. 42.7 ppm because some AuNPs were adsorbed onto the ceramic rods. Therefore, the calculated quantity of AuNPs on each ceramic rod was ca. 7.3 × 10^−5^ g (0.2 L × (50 − 42.7) ppm(mg L^−1^)/20). Then the AuNP-coated ceramic rods were rinsed thoroughly with deionized (DI) water, and finally dried in an oven at 120 °C for 1 day. Before preparing the PAW solutions, the AuNP-coated ceramic rods were immersed and rinsed with DI water for several cycles until the pH values of the DI water were almost identical (ca. pH 7.15 and water temperature at ca. 24 °C) before and after the rinsing process.

### Preparation of the PAW solutions in situ and ex situ in sunshine

Eighteen AuNP-coated ceramic rods were placed in sample vials containing 250 mL of 0.1 M KCl-containing DI water solutions. Then the sealed sample vials were placed in direct natural sunshine for 3 h around noon (from ca. 10:30 to 13:30) to create the PAW solutions in situ. After this solar irradiation, the pH of the solution slightly changed from 7.15 to 7.21, and the temperature of the solution increased from 24 to 35 °C. The first examination of the created PAW solution in situ was immediately performed after the solution had been naturally cooled down to room temperature. Generally, this cooling process took less than 2 h after solar irradiation. Because all of the experiments based on the prepared PAW were performed at room temperature the created warm PAW under sunshine was further cooled down to room temperature. The PAW solution ex situ was created by following a similar process for creating the PAW solution in situ, but no electrolyte was added to the DI water before preparation of PAW in the sunshine. Instead, KCl was added to the created PAW immediately after removal from the sunshine to prepare the 0.1 M KCl-containing PAW solution ex situ. Similarly, the first examination of the created PAW solution ex situ was immediately performed after the solution had been naturally cooled down to room temperature. To examine the purity of the prepared PAW solution, further inductively coupled plasma-mass spectrometer (ICP-MS) analyses indicated that the concentrations of dissolved metals in the PAW solution after irradiation were ca. 0.48, 23, 14, 17, 7.6, and 2.3 ppb for Au, Na, K, Al, Mg, and Ca, respectively. These values were ca. 0.22, 19, 15, 21, 10, and 3.6 ppb for Au, Na, K, Al, Mg, and Ca, respectively, for a similar experiment performed in the dark as a reference. The increased concentration of Au is from the dissolved AuNPs on the AuNPs-coated ceramics due to hot electron transfer after direct sunlight^21^. The differences in concentrations of other metals are from the measuring fluctuations due to the low concentrations on ppb levels.

## Supplementary information

Supplementary Information.
