# Supplementary material for: New solar energy-storage resource of plasmon-activated water solution with higher chemical potential
Source: Sci Rep. 2020 Nov 30;10:20868. doi: 10.1038/s41598-020-77815-3 (PMC7705734; doi:10.1038/s41598-020-77815-3)
Supplement: Supplementary file 1 — Supplementary Information. [file 41598_2020_77815_MOESM1_ESM.docx]

**Supporting Information**

**New Solar Energy-Storage Resource of Plasmon-Activated Water Solution with Higher Chemical Potential**

Chih-Ping Yang^1,#^, Shih-Hao Yu^1, #^, Fu-Der Mai^1^, Tai-Chih Kuo^1,*^, Yu-Chuan Liu^1,2*^

^1^Department of Biochemistry and Molecular Cell Biology, School of Medicine, College of Medicine, Taipei Medical University, No. 250, Wuxing St., Taipei 11031, Taiwan

^2^Cell Physiology and Molecular Image Research Center, Wan Fang Hospital, Taipei Medical University, Taipei, Taiwan

^*^ Corresponding authors at: [tckuo@tmu.edu.tw](mailto:tckuo@tmu.edu.tw); 886-2-27361661, ext 3167 (T.-C. K.) and [liuyc@tmu.edu.tw](mailto:liuyc@tmu.edu.tw); 886-2-27361661, ext 3155 (Y.-C. L.)

^#^ C.-P. Y. and S.-H. Y. contributed equally to this work.

**Methods**

**Chemicals and materials.** The NaCl electrolyte was purchased from Sigma-Aldrich Organics. The KCl and NaOH electrolytes, and the K_3_Fe(CN)_6_ reagent were purchased from Acros Organics. All of the reagents were used as received without further purification. Commercial chitosan (Ch) powder with an 0.82 degree of deacetylation was purchased from First Chemical Works, Taiwan. Ceramic rods (with molar compositions of 92% SiO_2_, 3.0% Na_2_O and K_2_O, 2.0% Fe_2_O_3_, 1.5% Al_2_O_3_, 0.5% CaO and MgO, and other rare metal oxides) were purchased from Chyuan-Bang Enterprise, Taiwan. All of the solutions were prepared using deionized (DI) water at 18.2 MΩ·cm provided by a Milli-Q system. Before prepare the DI water the used water is first treated by reverse osmosis (RO) to prepare ultrapure water. All experiments were performed in an air-conditioned room at ca. 25 °C. The water temperature was ca. 24 °C.

**Preparation of gold nanoparticles (AuNPs)**. The AuNPs (ca. 10 nm) in an aqueous solution were obtained from an Au sheet (with a purity of 0.9999) using electrochemical and thermal reduction methods, as shown in our previous report^1^. Typically, an Au electrode was cycled in a deoxygenated aqueous solution of 40 mL containing 0.1 N NaCl and 1 g L^-1^ Ch from -0.28 to +1.22 V vs. Ag/AgCl at 500 mV s^-1^ for 200 scans under slight stirring. Durations at the cathodic and anodic vertices were 10 and 5 s, respectively. Immediately, without changing the electrolytes, the solution was heated from room temperature to boiling at a heating rate of 6 °C min^-1^ in air. After cooling, the clear AuNP-containing solution was separated from the settled Ch. Then the AuNP-containing solution was placed in an ultrasonic bath for 30 min and was further centrifuged at 3600 rpm for 2 min to remove the Ch to prepare pure AuNPs in solution.

**Evaporation rate of the PAW solutions at room temperature into ambient laboratory air.** Samples of 10 mL of PAW solutions were added to open glass sample bottles (20 mL), which were placed on a platform of an orbital shaker, operated at 150 rpm at room temperature. The weight of each glass sample bottle, containing a solution, was measured every a 0.5 h for 1 h to determine the evaporating mass (mg) of water per half hour. In these experiments, the relative humidity (RH) was 47%, and the room temperature was 25 °C. The evaporation mass was determined by the whole mass (including water and glass bottle) at a specific time minus the whole mass before the specific time. Replicate measurements based on three similar samples were performed. Errors were obtained from the relative standard deviation (RSD).

**Heating process of a PAW solution to obtain its specific heat compared to a DI water solution.** 100 g PAW solution *in situ* containing 0.1 M KCl (or similar DI water solution) was added in a glass beaker (250 mL). Then the opening of the glass beaker was covered with wrap and the solution-containing glass beaker was heated from room temperature to boiling point with a heater at a fixed heating rate (Corning, pc-420D) in ambient laboratory air. The temperature of the solution was recorded with a temperature meter (First clean corporation, pH 500). Mass losses at the boiling point were 6.22, 7.16, 7.10, and 6.40 g for the DI water solution, the PAW solution *in situ*, the PAW solution *ex situ* and a blank solution, respectively.

**Nuclear magnetic resonance (NMR) relaxation time measurements**. NMR-T_1_ values of the PAW solution *in situ* and DI water solution were measured at atmospheric pressure using a Bruker Fourier 300 spectrometer, operating at a ^1^H resonance frequency of 300 MHz. For the measurement, a repetition time of 10 s and acquisition of eight signal averages were employed for each delay time. The data were analyzed with the help of a Bruker T_1_ analytical routine with a software package.

**Electrochemical oxidation-reduction cycles (ORCs) on the Au substrate**. Electrochemical experiments were performed in a three-compartment cell at room temperature (25 °C) and were controlled by a potentiostat (model PGSTAT30, Eco Chemie). A gold sheet with a bare surface area of 0.238 cm^2^, a 1 × 4-cm platinum sheet, and a KCl-saturated silver-silver chloride (Ag/AgCl) electrode were respectively employed as the working, counter, and reference electrodes. Before ORC treatment, the gold electrode was mechanically successively polished (model Minimet 1000, Buehler) with 1- and 0.05-µm alumina slurries to a mirror finish. Then the electrode was cycled in a deoxygenated PAW solution *in situ* (40 mL containing 0.1 M KCl) or in a deoxygenated DI water solution (35 mL containing 0.1 M KCl) from -0.28 to +1.22 V vs. Ag/AgCl at 500 mV s^-1^ for 20 scans. Respective durations at the cathodic and anodic vertices were 10 and 5 s. Finally, the potential was held at the cathodic vertex before the roughened Au electrode was taken from the solution and thoroughly rinsed with DI water. These ORC procedures are generally employed in SERS studies in our^2^ and other laboratories^3,4^.

**Hydrogen evolution reactions (HERs) and oxygen evolution reactions (OERs)**. HERs or OERs were performed by linear sweep voltammetry (LSV) in a three-electrode system consisting of a Pt electrode (0.238 cm^2^), a Pt sheet, and a KCl-saturated Ag/AgCl electrode as the working, counter, and reference electrodes, respectively. Typically, the corresponding electrochemical measurement was carried out in a deoxygenated 35-mL PAW solution *in situ* (or DI water solution) with 0.1 M KCl at a scan rate of 0.05 V s^–1^. These electrochemical experiments were also controlled by a potentiostat (model PGSTAT30, Eco Chemie).

**Additional Discussions**

**Electron transfer rate constant (ks)**. As shown in Figs. S3, S4, and S5, as the scan rate (υ) increased from 50 to 600 mV s^–1^, the redox peak currents of Fe(CN)_6_^3–/4–^ simultaneously increased. The low-conductivity solution, which cannot instantly respond at high υ values because of the slow electron transfer rate in the absence of electrolytes, resulted in more-positive and negative shifts in the anodic peak potential (E_pa_) and cathodic peak potential (E_pc_), respectively, as υ increased. Furthermore, when the anodic peak-to-cathodic peak separation was >0.2 V and υ was >200 mV s^–1^, the peak potentials were proportional to the natural logarithm of υ (plots c and d of Figs. S3, S4 and S5). Two linear equations (most of which with R^2^ values of >0.999) were obtained for the anodic and cathodic peak potentials. According to equations (1) and (2)^5^:

$E_{pa}=E^{0^{'}}-\frac{RT}{\left( 1-\alpha\right)nF}\ln\frac{{RTk}_{S}}{\left( 1-\alpha\right)nF}+\frac{RT}{\left( 1-\alpha\right)nF}ln \upsilon$(1)

$E_{pc}=E^{0^{'}}+\frac{RT}{\alpha nF}\ln\frac{{RTk}_{S}}{\alpha nF}-\frac{RT}{\alpha nF}ln \upsilon$ (2)

where E^0’^ is the formal potential (i.e., the average of E_pa_ and E_pc_), α is the electron transfer coefficient, n is the number of electrons transferred, T is the absolute temperature, R is the gas constant, F is Faraday’s constant, *k_a_* and *k_c_* are the slopes of anodic and cathodic peaks potentials, respectively, and vs ln υ. *k_s_* is the apparent heterogeneous electron transfer rate constant. The n value of Fe(CN)_6_^3–/4–^ was 1. Therefore, *k_s_* of K_3_Fe(CN)_6_ in PAW solution *in situ* (0.233±0.014 s^−1^) and in PAW solution *ex situ* (0.226±0.015 s^−1^) are ca. 6.9% and 3.7%, respectively, higher than that in DI water solution (0.218±0.014 s^−1^). Also, these constants for PAW solution *in situ* (0.257±0.008 s^−1^) and for PAW solution *ex situ* (0.233±0.005 s^−1^) are ca. 18% and 7.4%, respectively, still higher than that for DI water solution (0.217±0.005 s^−1^) after the samples had aged for 2 days.

**References**

1. Yu, C. C. Liu, Y. C., Yang, K. H. & Tsai, H. Y. Simple method to prepare size-controllable gold nanoparticles in solutions and their applications on surface-enhanced Raman scattering. *J. Raman Spectrosc*. **42**, 621−625 (2011).

2. Liu, Y. C., Hsu, T. C. & Tsai, J. F. Thermal stability of electrochemically prepared surface-enhanced Raman scattering-active metals substrates. *J. Phys. Chem. C* **111**, 10570–10574 (2007).

3. Wang, C. C. Surfaced-enhanced Raman scattering-active substrates prepared through a combination of argon plasma and electrochemical techniques. J. Phys. Chem. C **112**, 5573–5578 (2008).

4. Mosier-Boss, P. A. & Lieberman, S. H. Surface-enhanced Raman spectroscopy (sers) and molecular modeling of the chromate interaction with 4-(2-mercaptoethyl)pyridinium. *Langmuir* **19**, 6826–6836 (2003).

5. Laviron, E. Autoinhibition and autocatalysis in polarography and in linear potential sweep voltammetry. *J. Electroanal. Chem*. **52**, 355–393 (1974).


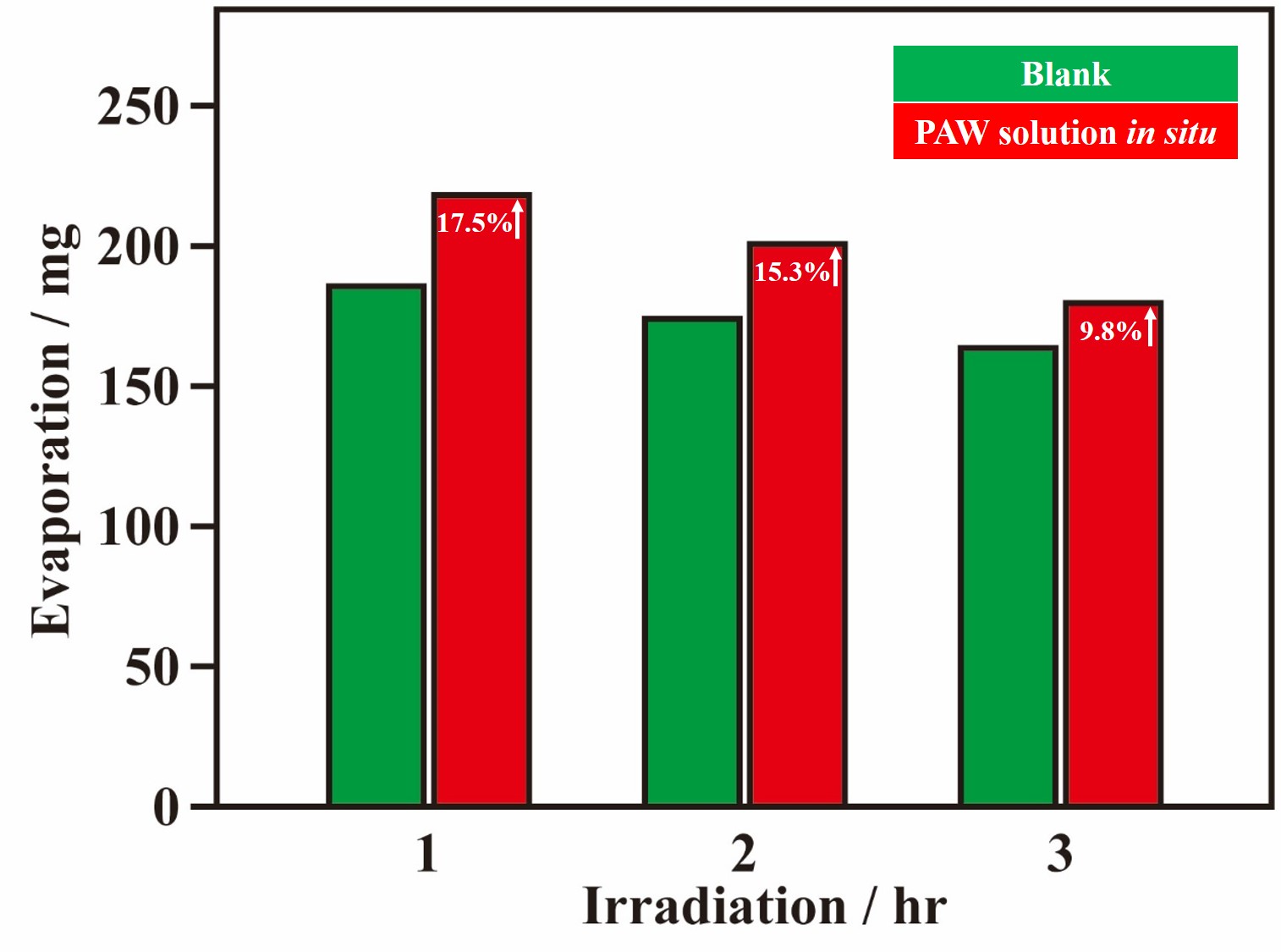


Fig. S1. Evaporation rates as a function of the irradiation time of PAW solution (0.1 M KCl) *in situ* and blank experiment-based solution (0.1 M KCl) under exposure to sunlight for 3 hr; the blank solution was obtained by using experimental conditions similar to those for preparing the PAW solution *in situ* but using blank ceramic rods with no AuNP coating. In experiments, six AuNP-coated ceramic rods were placed in glass sample bottles containing 50 mL of 0.1 M KCl-containing DI water solutions. Then the open bottles were placed in sunshine for 3 hr around noon to create the PAW solutions *in situ*. The weight of each bottle, containing a solution, was measured every one hour for 3 hrs to determine the evaporating mass (mg) of water per hour. The measured evaporation masses in the first hour were 185.30±12.99 and 218.13±14.21 mg for the blank solution and the PAW solution *in situ*, respectively. The measured evaporation masses in the second hour were 174.07±11.95 and 200.73±10.10 mg for the blank solution and the PAW solution *in situ*, respectively. The measured evaporation masses in the third hour were 163.60±10.84 and 179.67±8.44 mg for the blank solution and the PAW solution *in situ*, respectively.


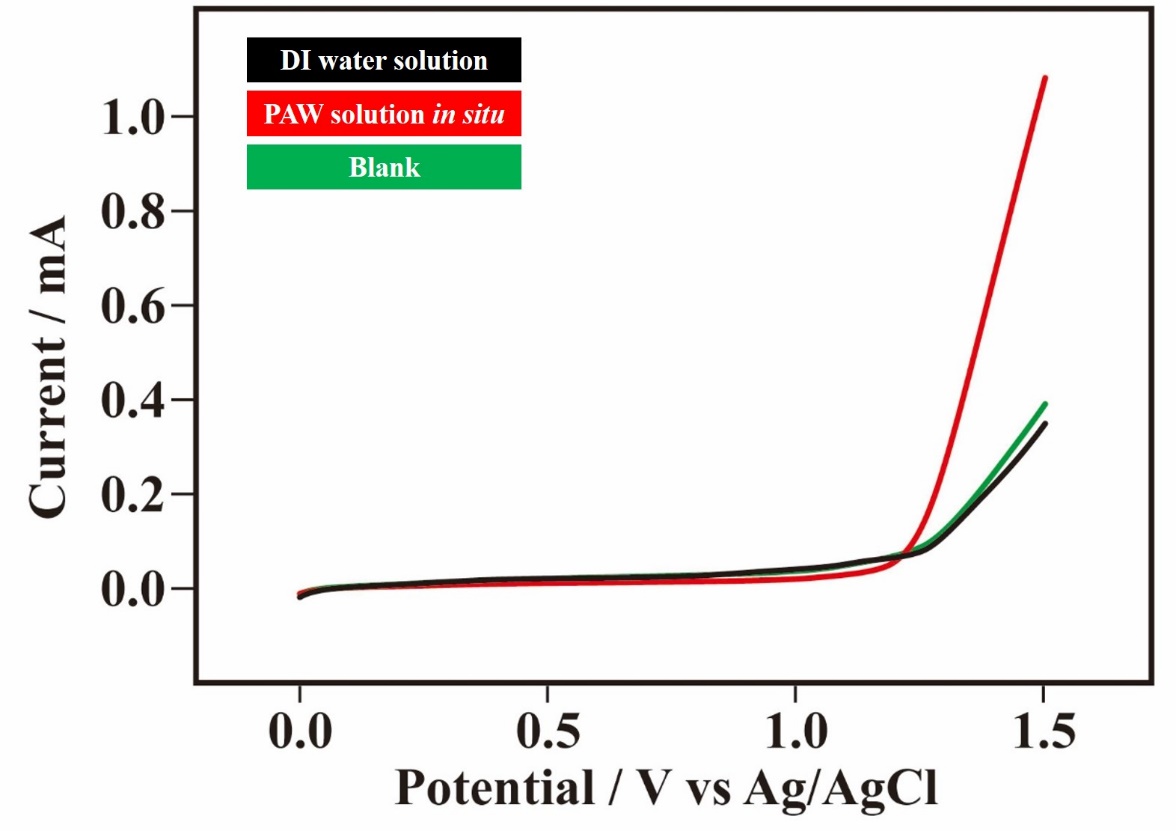


Fig. S2. Linear sweep voltammetry (LSV) at scan rates of 0.05 V s^–1^ recorded on a planar Pt electrode for oxygen evolution reaction (OER) in PAW solution *in situ*, DI water solution and blank solution (all containing 0.1 M KCl). The recorded currents were 1.027±0.138, 0.332±0.050 and 0.363±0.085 mA at vertex of 1.5 V for PAW solution *in situ*, DI water solution and blank solution, respectively. The blank solution was obtained by using the similarly experimental conditions as preparation of PAW *in situ* solution but employing the blank ceramic rods without coating of AuNPs.


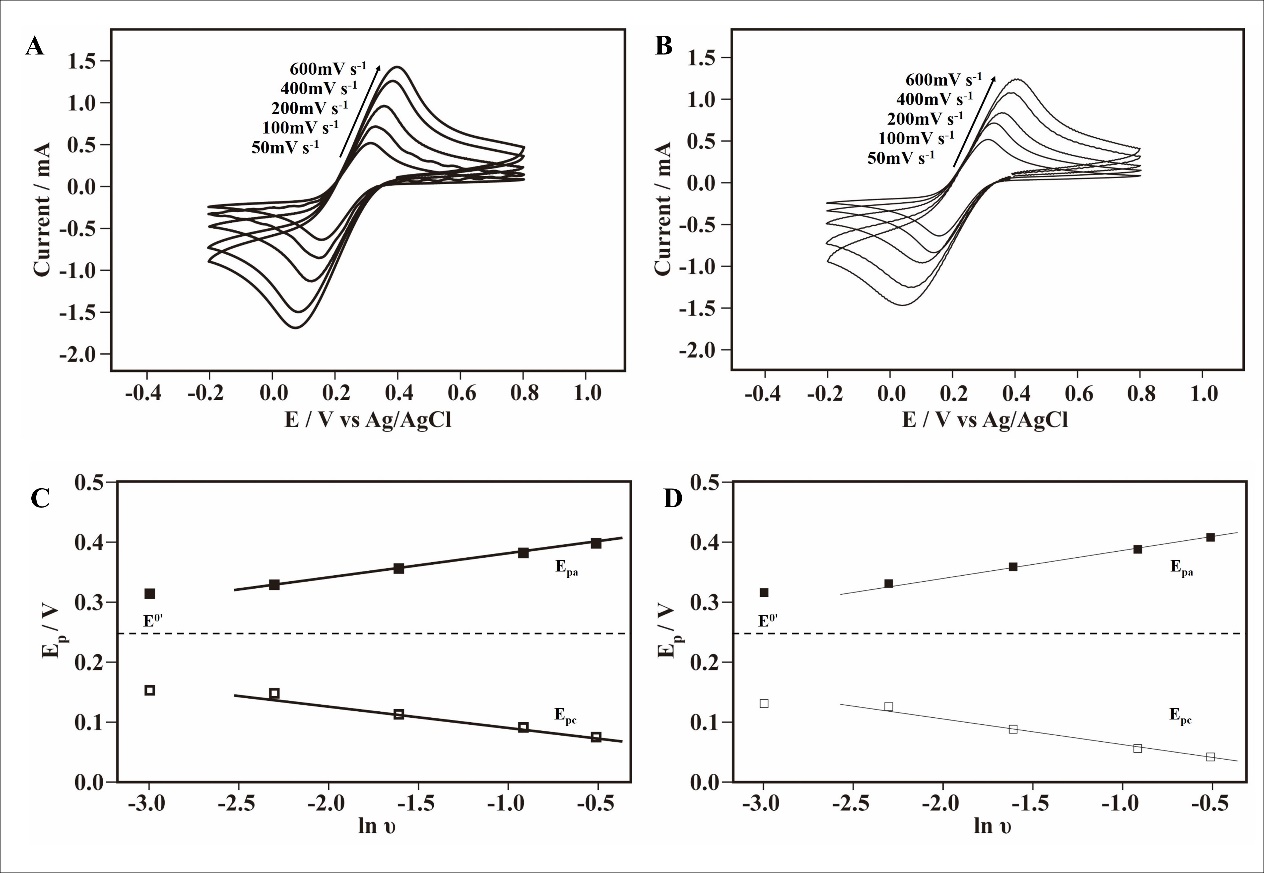


Fig. S3. Cyclic voltammograms (CV) recorded on the same planar Au electrode showing the third scans in oxidation-reduction cycle (ORC) at various scan rates υ (50~600 mV s^–1^) in as-prepared and aged (for 2 days) DI water solutions (50 mM K_3_Fe(CN)_6_). (a) As-prepared DI water solution. (b) Aged DI water solution. (c) Plots of anodic peak potential (E_pa_) and cathodic peak potential (E_pc_) vs ln υ from plot (a). (d) Plots of anodic peak potential (E_pa_) and cathodic peak potential (E_pc_) vs ln υ from plot (b).


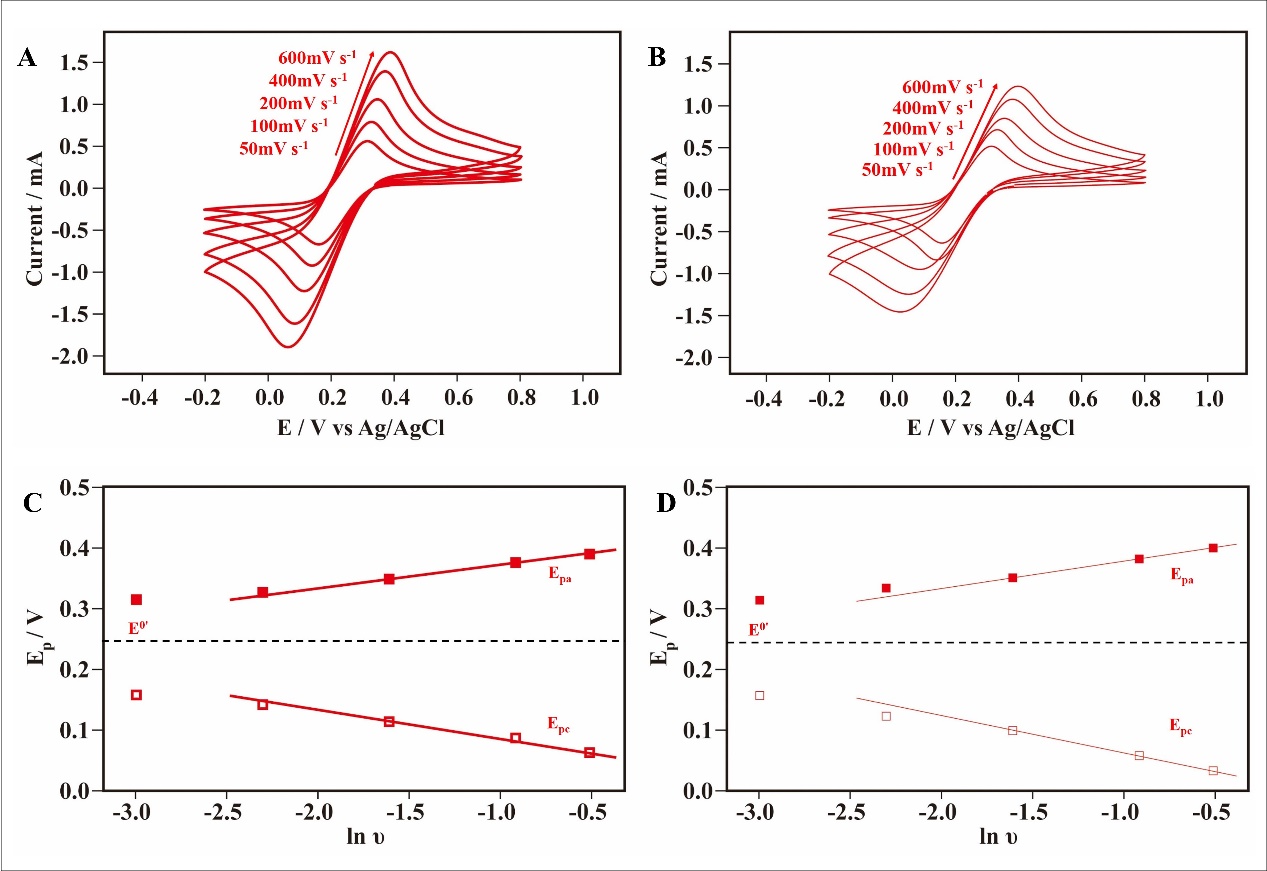


Fig. S4. Cyclic voltammograms (CV) recorded on the same planar Au electrode showing the third scans in oxidation-reduction cycle (ORC) at various scan rates υ (50~600 mV s^–1^) in as-prepared and aged (for 2 days) PAW solutions *in situ* (50 mM K_3_Fe(CN)_6_). (a) As-prepared PAW solution *in situ*. (b) Aged PAW solution *in situ*. (c) Plots of anodic peak potential (E_pa_) and cathodic peak potential (E_pc_) vs ln υ from plot (a). (d) Plots of anodic peak potential (E_pa_) and cathodic peak potential (E_pc_) vs ln υ from plot (b).


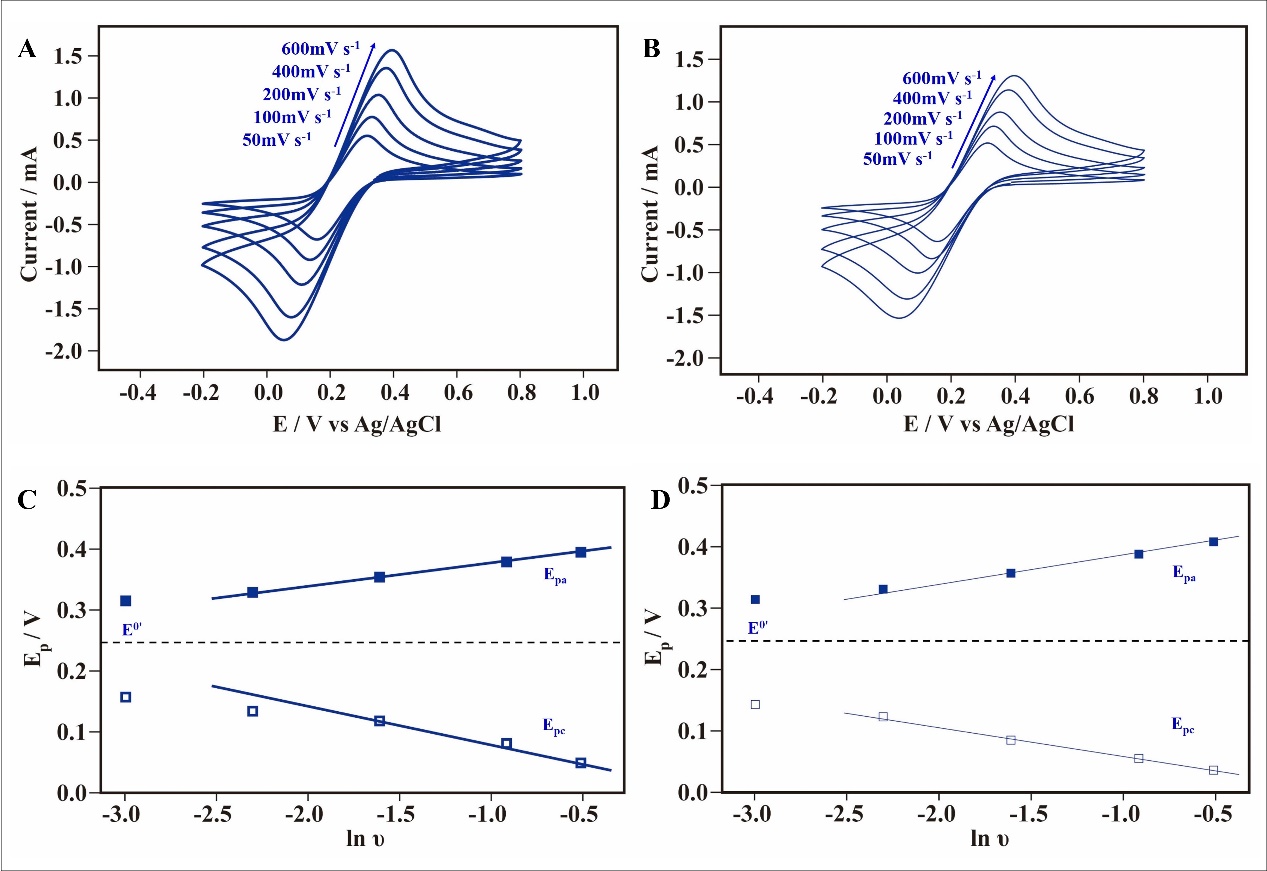


Fig. S5. Cyclic voltammograms (CV) recorded on the same planar Au electrode showing the third scans in oxidation-reduction cycle (ORC) at various scan rates υ (50~600 mV s^–1^) in as-prepared and aged (for 2 days) PAW solutions *ex situ* (50 mM K_3_Fe(CN)_6_). (a) As-prepared PAW solution *ex situ*. (b) Aged PAW solution *ex situ*. (c) Plots of anodic peak potential (E_pa_) and cathodic peak potential (E_pc_) vs ln υ from plot (a). (d) Plots of anodic peak potential (E_pa_) and cathodic peak potential (E_pc_) vs ln υ from plot (b).
